# miRNAs as Novel Biomarkers of Chronic Kidney Injury in Anabolic-Androgenic Steroid Users: An Experimental Study

**DOI:** 10.3389/fphar.2020.563756

**Published:** 2020-09-16

**Authors:** Francesco Sessa, Monica Salerno, Giuseppe Bertozzi, Luigi Cipolloni, Giovanni Messina, Mariarosaria Aromatario, Lorenzo Polo, Emanuela Turillazzi, Cristoforo Pomara

**Affiliations:** ^1^ Department of Clinical and Experimental Medicine, University of Foggia, Foggia, Italy; ^2^ Department of Medical, Surgical and Advanced Technologies “G.F. Ingrassia”, University of Catania, Catania, Italy; ^3^ Department of Anatomical, Histological, Forensic and Orthopedic Sciences, Sapienza University of Rome, Rome, Italy; ^4^ Brain srl, Services and Consultancy in Health, Pavia, Italy; ^5^ Translational Research and New Technologies in Medicine, University of Pisa, Pisa, Italy

**Keywords:** anabolic androgenic steroids, kidney injury, miRNAs, miRNA dysregulation, new molecular biomarkers

## Abstract

miRNAs are a family of 20–22 non-coding nucleotides that control gene expression by inhibiting the translation of their target messenger RNAs (mRNAs). Two models have been proposed to elucidate the mechanism of action: they act either hindering mRNA translation or enhancing mRNA degradation. Anabolic-Androgenic Steroids (AASs) represent a class of drugs used to treat several diseases. In the last few years, AASs have frequently been used for aesthetic purposes, indeed, they form part of the larger group called image- and performance-enhancing drugs (IPEDs). Long-term AAS use can lead to serious health consequences. In this regard, the present study aimed to analyze the role of several microRNAs (miRNAs) in renal damage after AAS use, to better understand the underlying mechanisms. For this purpose, two miRNAs (miR-21 and miR-205) were tested in two groups: AAS group (seven males, mean age 33.28 ± 4.68 years; mean body mass index (BMI) 27.04 ± 1.07), and chronic kidney disease (CKD) group (seven males, mean age 66.2 ± 5.4 years; mean BMI 24.75 ± 1.35). Finally, the same miRNAs were tested in the “Control” group (seven males, mean age 44.85 ± 5.75 years; mean BMI 26.5 ± 1.88). Kolmogorov-Smirnov Test was used to determine the normality of data distribution. All variables were normally distributed. Student’s t-test was used for comparisons between two groups. Analyzing the results of the present study, the two tested miRNAs (miR-21 and miR-205) were significantly higher in the CKD group compared to the AAS group, with mir-21 being much more expressed than miR-205. This study represents a pilot study to define if these expression patterns could be studied in other biological samples (plasma, urine) in subjects with different kidney injury linked to chronic kidney diseases and AAS use, to identify reliable biomarkers that could be applied in clinical and forensic diagnostics, as well as a target for toxicological investigations or therapeutic treatments.

## Introduction

miRNAs are a family of 20–22 non-coding nucleotides that control gene expression by inhibiting the translation of their target messenger RNAs (mRNAs) (O’Brien et al., 2018). The first miRNAs were identified about 20 years ago and since their discovery a pivotal role in post-transcriptional mRNA regulation at the cytoplasm level has been described. Two models have been proposed to elucidate the mechanism of action: they act either hindering mRNA translation or enhancing mRNA degradation (Catalanotto et al., 2016). In consideration of their fundamental functions, in the last few decades, they have been investigated as the main goal of a large number of scientific studies with the following aims: on the one hand to identify the underlying altered pathway, focusing on the modification of their expression in cases of pathology (Sessa et al., 2019); on the other hand, in cases of a specific disease, to ascertain if miRNAs could be involved, becoming both diagnostic markers and, possibly, therapeutic targets (Sessa et al., 2018b; Sessa et al., 2020).

Anabolic-Androgenic Steroids (AASs) represent a family of drugs used to treat several diseases, such as aging, cancer, and AIDS (Sawant et al., 2014; Ip et al., 2015), or in cases of delayed puberty (Hartgens and Kuipers, 2004; Pomara et al., 2014; Albano et al., 2017; Christou et al., 2017; Goldman and Basaria, 2018). In the last few years, AASs have frequently been used for aesthetic purposes, indeed, they form part of the larger group called image- and performance-enhancing drugs (IPEDs). For these reasons, their use is not limited to athletes, but involves a large number of young people (De et al., 2015; Sessa et al., 2018a; Hearne et al., 2020). The results of a recent meta-analysis highlighted that the nonmedical AAS use represents a major global public health problem that requires the attention of policymakers and researchers. Particularly, AAS epidemiology and prevalence are higher in Europe, the Middle East, North America (the USA), Oceania (Australia and New Zealand), and South America (Brazil) and lower in Africa and Asia (Sagoe et al., 2014). Some studies assessed the prevalence of AAS use among resistance training practitioners in gyms, resulting in a prevalence range of 4.5 to 24.9% (Pellegrini et al., 2017). Moreover, this kind of drug is commonly used by other categories such as recreational sportspeople, and sexual and gender minorities (Sagoe and Pallesen, 2018).

**Figure 1:**
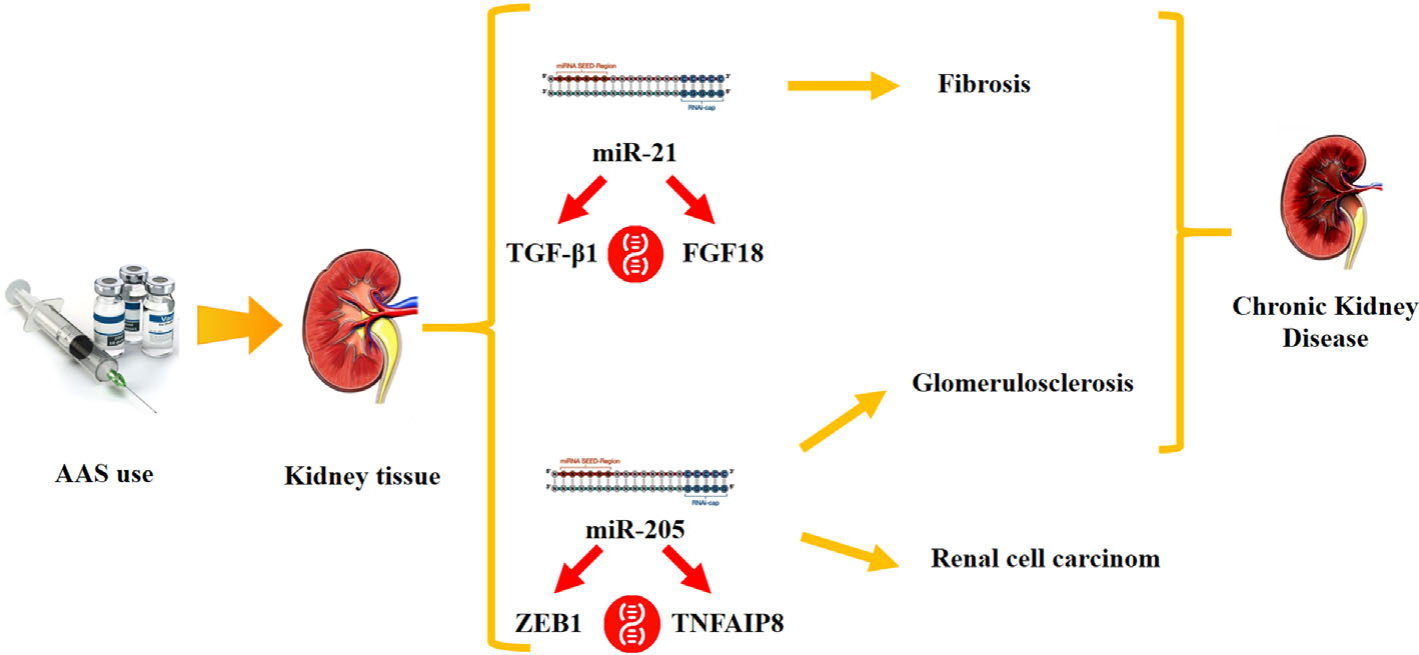
Our study hypothesis: Anabolic-Androgenic Steroid (AAS) use may cause a miRNA dysregulation, generating kidney damage. Testing the expression values of several miRNAs involved in kidney function (miR-21 and miR-205), this study aimed to highlight the differences of these molecular biomarkers among the two tested groups, AAS group and “Chronic Kidney Disease” group.

Long-term AAS use can lead to serious health consequences (Bertozzi et al., 2019c), generating different human hormonal diseases such as severe acne, development of breasts in men (gynecomastia) (Piacentino et al., 2014), facial and body hair growth in women (hirsutism) (NIDA, 2018), shrinkage of testicles (Pomara et al., 2016; Barone et al., 2017; NIDA, 2018), baldness in both sexes, irregular menstruation, and infertility (De Souza and Hallak, 2011); additionally, AAS use may generate yellowing of the eyes or skin (jaundice), high blood pressure (Riezzo et al., 2011), neuropsychiatric disorders (Bertozzi et al., 2017; Bertozzi et al., 2019b), and liver tumors or other cancers (Salerno et al., 2018; Agriesti et al., 2020). Furthermore, a recent paper reviewed kidney injury related to AAS use/abuse, highlighting the important role in the generation of both acute and chronic renal lesions (Parente Filho et al., 2020). In this regard, the present study aimed to analyze the role of several microRNAs (miRNAs) in renal damage after AAS use, to better understand the underlying mechanisms. For this purpose and after a literature review, two miRNAs were selected, the expression of different types of kidney damage: miR-21 and miR-205. miR-21 has been shown to act on epithelial cells and myofibroblasts promoting the development of fibrosis, which is crucial in chronic kidney disease (CKD) (Lorenzen et al., 2011; Gomez et al., 2013). On the other hand, miR-205 is involved in the transition from epithelial to renal mesenchymal cells, significantly suppressed in renal cancer cell (RCC) samples and markedly overexpressed in cases of hypertensive glomeruloscleroses; the degree of upregulation is directly proportional with disease severity (Schena et al., 2014) (**Figure 1**).

## Materials and Methods

### Selected Cases

All samples were selected analyzing the autopsy documentation of cases examined by the Institute of Legal Medicine of Foggia from 2001 to September 2019 (about 1,700 autopsies). All procedures were performed in accordance with the Declaration of Helsinki and were approved by the Scientific Committee of the University of Foggia. Seven cases of young men (29–40 years), with a post-mortem toxicological positive test for anabolic agents, were selected (mean age 33.28 ± 4.68 years; mean body mass index (BMI) 27.04 ± 1.07). Particularly, in two cases, the victims tested positive for synthetic testosterone; nandrolone and synthetic testosterone were found in three cases; finally, nandrolone was detected in the last two cases. These samples made up the AAS group. Seven cases of men who had died of cardiac arrest after a long period (from 2 to 5 years) of Chronic kidney disease (CKD) were selected (mean age 66.2 ± 5.4 years; mean BMI 24.75 ± 1.35), making up the CKD group. Finally, seven cases of healthy men without neurological diseases (mean age 44.85 ± 5.75 years; mean BMI 26.5 ± 1.88), who had died in car accidents were selected as reference samples in the Real-Time PCR reactions (“Control” group).

### miRNA Selection

The databases Medline, Scopus, Web of Science, and Google Scholar were searched from January 2007 to December 2019, using the following keywords: “Kidney Injury”, “Kidney Damage”, “miRNA”, “miRNA dysregulation”, and “Anabolic Androgenic Steroids”. The main keywords, “Kidney Injury” and “miRNA”, were searched for in association with each of the others. At the end of the literature review, the following miRNAs were selected: hsa-miR-21-5p, and hsa-miR-205-5p.

### miRNA Quantitative Real-Time PCR (qRT-PCR)

The Recover All Total Nucleic Acid Isolation Kit (Life Technologies) was used to obtain total RNA working from formaldehyde-fixed paraffin-embedded (FFPE) kidney samples (four 20µm sections) as previously described (Sessa et al., 2019). To quantify the obtained RNA, the Qubit Fluorometer with the Qubit RNA HS Assay Kit (Life Technologies) was used. To obtain the miRNA profiling of the selected miRNAs (hsa-miR-21-5p, and hsa-miR-205-5p) the TaqMan Advanced miRNA Assay (ThermoFisher Scientific) kit was used. This kit is composed of a pre-formulated primer and probe set. Following the kit protocol, cDNA was obtained running the samples in the StepOnePlus Real-Time PCR System (ThermoFisher Scientific), analyzing the raw data with the relative software (version 2.3). The expression value data were analyzed following the method of normalization with the endogenous control, selecting the Assay from among those suggested by the producer. For this experimental study, to consolidate the results, two endogenous controls were selected: hsa-miR-186-5p and hsa-miR-361-5p (TaqMan Advanced miRNA Assays, ThermoScientific).

Expression fold changes were computed using the 2^−ΔΔCt^ calculation (Livak and Schmittgen, 2001), where ΔCt = Ct(test miRNA) − Ct(mir-186-5p) and ΔΔCt = ΔCt(individual sample) − ΔCt (control median samples).

### Statistics

Descriptive statistical analyses were performed using different software packages (Microsoft Office Excel 2007, StataIC, StataCorp and R). Kolmogorov-Smirnov Test was used to determine the normality of data distribution. All variables were normally distributed. Student’s t-test was used for comparisons between two groups. P < 0.05 was considered to represent a statistically significant difference.

## Results

The quantitative analysis evaluated the expression levels of miRNA hsa-miR-21-5p, and hsa-miR-205-5p, in the AAS group and the CKD group. The values of the “Control” group were used to apply the ΔΔCT method. In **Table 1**, the results of the expression values of each miRNA tested, subdivided for each group, are summarized. The expression values of each tested samples are summarized in the supplementary file (**Table S1**).

**Table 1:** Expression levels of miRNAs analyzed in each group.

	Mean Expression levels(endogenous control: miR-186)		Mean Expression levels(endogenous control: miR-361)	
	miR-21-5p	miR-205-5p	miR-21-5p	miR-205-5p
AAS (Mean values)	1.65 ± 0.74	0.77 ± 0.51	1.8 ± 0.55	0.76 ± 0.46
CKD (Mean values)	2.83 ± 0.74	1.64 ± 0.78	2.84 ± 0.61	1.72 ± 0.76

**Figure 2:**
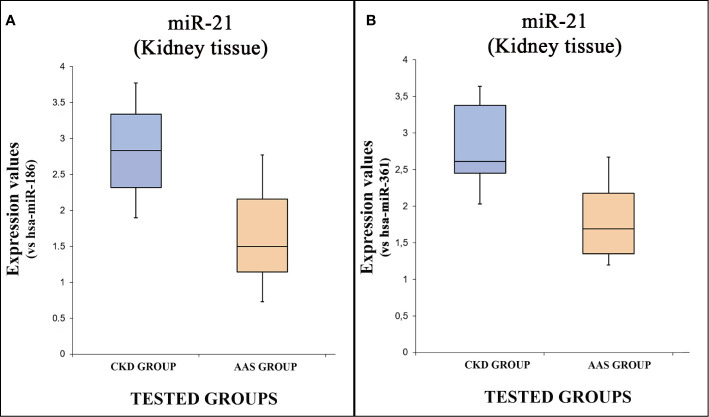
The box plot analysis compares the expression levels of hsa-miR-21-5p in each group using two endogenous controls [has-miR-186, **(A)**; has-miR-205, **(B)**].

Moreover, a statistical analysis was performed analyzing the expression values of each miRNA tested, comparing all groups. The expression values of hsa-miR-21-5p are summarized by box plot analysis, using two endogenous controls. In **Figure 2A**, the plot refers to the values obtained using has-miR-186 as endogenous control, while **Figure 2B**, shows the plot related to the has-miR-361 endogenous control.

There were statistically significant differences among groups as determined using both miR-186 [p = <0.05(0.009)] and miR-361 as endogenous controls [p = <0.05(0.002)]: the expression level of hsa-miR-21-5p was significantly higher in the CKD group compared to the “AAS” group.

**Figure 3:**
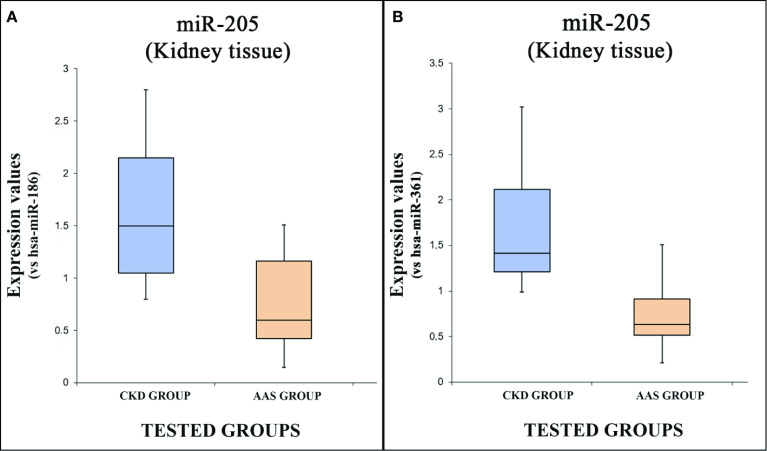
The box plot analysis compares the expression levels of hsa- miR-205-5p in each group using two endogenous controls [has-miR-186; **(A)** has-miR-205, **(B)**].

The expression values of hsa-miR-205-5p are summarized using the box plot analysis (**Figures 3A, B**, using miR-186 and miR-361 as endogenous controls).

There were statistically significant differences between the two groups as determined by Student’s t-test both using miR-186 as endogenous control [p = <0.05(0.03)] and miR-361 [p = <0.05(0.007)].

## Discussion

The adverse effects of AASs on the kidney were recently analyzed by Davani-Davari et al. (2019) through a literature review. These authors suggested that AASs assumption can affect the kidney in different ways, inducing or aggravating acute kidney injury, chronic kidney disease, and glomerular toxicity. Although a dose-related nephrotoxic effect has been proposed (D’Errico et al., 2011), to the best of our knowledge, no studies have been published about miRNAs dysregulation, kidney damages, and AASs use.

The present study demonstrates that the different investigated situations, AAS use and CKD, caused miRNA dysregulation at the kidney level. The tested miRNAs (miRNA hsa-miR-21-5p, miR-205) were overexpressed in both groups tested (AAS and CKD groups) compared to the control. These results could explain what was found by El-Reshaid et al. (2018). The chronic use of AAS combined with a high-protein diet can generate severe renal damage such as focal segmental glomerulosclerosis (FSGS), nephroangiosclerosis, chronic interstitial nephritis, and acute interstitial nephritis. In a recent case report, Garcia et al. described acute kidney failure in a bodybuilder who had taken intramuscular anabolic-androgenic steroids (testosterone and stanozolol) (Merino García et al., 2018). Another study reported that FSGS had been frequently detected in AAS users, suggesting two mechanisms of action: direct glomerular toxicity and glomerular hyperfiltration related to the increased body mass of abusers (Herlitz et al., 2010). Moreover, renal side effects have also been described leading to acute renal failure and even Wilms’ tumors in isolated cases (Modlinski and Fields, 2006). A direct relationship between AAS use and kidney injury has been described in several animal studies. High levels of several cytokines (such as TNF-α) (Patil et al., 2016) and hypertension can cause the development of glomerulosclerosis (Doublier et al., 2011), generating injuries similar to CKD (Loh et al., 2017). CKD is caused by the progressive development of glomerular fibrosis or glomerulosclerosis, interstitial fibrosis, consequent atrophy, loss of tubular epithelium, and chronic inflammation, also with the loss of the peritubular capillaries that causes a decrease in the glomerular filtration rate (Nogueira et al., 2017). In this way, and taking advantage of the ability of miRNA to be highly stable without changes generated by postmortem modification, they could be used as specific circulating and/or tissue biomarkers (Sessa et al., 2018b). These kinds of biomarkers could be very useful in the near future for anti-doping and toxicological purposes, or to identify kidney damage linked to chronic diseases, even in forensic practice (helping to define the overall clinical picture of a subject at the time of death, as well as helping to identify the exact cause of death).

Analyzing the results of the present study, the two tested miRNAs (miR-21 and miR-205) were significantly overexpressed in the CKD group compared to the AAS group, with miR-21 being much more expressed than miR-205. miRNA-21 is widely expressed in all tissues and is expressed quite highly in the normal kidney, heart, spleen, liver, and lung. Analyzing the target of this miRNA through Targetscan 7.2, miR-21 plays a pivotal role in the regulation of the transforming growth factor beta-induced (TGF-β1) gene. Its overexpression enhances TGF-β1-induced epithelial-to-mesenchymal transition by target smad7 and aggravates renal damage in diabetic nephropathy (Wang et al., 2014). Another gene target is fibroblast growth factor 18 (FGF18). It belongs to the FGF family that is involved in important biological and pathological processes, such as embryonic development, metabolic homeostasis and tumorigenesis through the regulation of cell differentiation, migration, proliferation and survival (Deng et al., 2020). As previously described, miR-21 was upregulated in injury with fibrosis both in mouse and human models (Chau et al., 2012). In addition to its involvement in kidney fibrosis (Zarjou et al., 2011; Zhong et al., 2011), several studies indicated that miR-21 may play an important role in stimulating fibrosis in other tissues after injury, such as cardiac and pulmonary tissues (Thum et al., 2008; Liu et al., 2010). Moreover, it has been reported that high levels of this miRNA promote kidney injury and fibrosis, while the inhibition of miR-21 in animal models ameliorates the effects (Chau et al., 2012). The way to regulate these mechanisms has been investigated in heart tissue: miR-21 regulates the ERK–MAP kinase signaling pathway in cardiac fibroblasts, impacting global cardiac structure and function. miR-21 levels are increased selectively in fibroblasts of the failing heart, augmenting ERK–MAP kinase activity through inhibition of sprouty homologue 1 (SPRY1). This mechanism regulates fibroblast survival and growth factor secretion, apparently controlling the extent of interstitial fibrosis and cardiac hypertrophy (Thum et al., 2008). It is commonly thought that the same pathway was followed for kidney tissue. Moreover, the action of this miRNA is exerted on PPARα as a major upstream regulator of the lipid metabolic signaling pathway (Lefebvre et al., 2006). PPARα has been identified as a protective transcription factor of the renal parenchyma. Its mechanism of action is expressed by the proliferation of peroxisomes that are used in the oxidation of fatty acids, also providing for the elimination of reactive oxygen species (ROS). In this way, the survival and functionality of renal epithelial cells, especially of the proximal tubules, but also of myofibroblasts, is guaranteed. Furthermore, miR-21 also inhibits acyl-CoA oxidase 1; acyl-CoA dehydrogenase and carnitine palmitoyl transferase, further worsening the redox state of the cell. In addition, miR-21 inhibits the Mpv17-like protein, a mitochondrial inner membrane protein identified in renal epithelial cells, capable of interacting through its PDZ domain with HtrA Serine Peptidase 2, which reduces the generation of ROS, thus preventing apoptosis (Gomez et al., 2013). Therefore, miR-21 represents a post-transcriptional regulator in kidney tissue that amplifies injury responses, resulting in increased fibrosis. In other words, it is possible to imagine that chronic use of AASs induces renal cellular stress, manifested by the increase in the expression of miR-21, so as to also stimulate the generation of ROS, and more generally fibrosis. All these mechanisms are the basis of CKD.

On the other hand, in the present study, the levels of miR-205 were overexpressed in both groups compared to controls. Even if the expression values were significantly higher in the CKD group compared to the AAS group. The target prediction program Targetscan 7.2 indicates that highly conserved binding sites for miR-205 are present in the Zinc Finger E-Box Binding Homeobox 1 (ZEB1) mRNAs. ZEB1 is a repressor of E-cadherin transcription that has been implicated in Epithelial mesenchymal transition (EMT) (Gregory et al., 2008). Moreover, this miRNA could be involved in the regulation of the Tumor necrosis factor-α-induced protein-8 (TNFAIP8) gene. Despite its pathophysiological function is not fully understood, this gene exerts several anti-apoptotic effects, promoting tumor development, invasion and metastasis: it is considered a vital factor participating in the process of cell survival and death (Zhang et al., 2018). Its overexpression, first of all, excludes the possibility that renal damage induced by the abuse of AAS could manifest itself through the development of renal cancer. In this case, miR-205 values would have been lower than controls (Schena et al., 2014; Ying et al., 2018). Conversely, Wang et al. reported that the expression levels of miR-205 in kidney tissue, similarly to other miRNAs investigated, were significantly higher in patients with hypertensive nephrosclerosis than controls (Wang et al., 2010). Moreover, a high intra-renal expression of miR-205 was also found in renal biopsies of patients with hypertensive glomerulosclerosis: these expression values were correlated with disease severity (Schena et al., 2014). The overexpression of miR-205, therefore, reinforces the hypothesis of the relationship between AAS-fibrosis-renal damage, however, this miRNA is linked to glomerular sclerosis alone, unlike miR-21, which includes a wider spectrum of fibroses, which also includes the promotion of myofibroblastic activity in response to increased oxidative stress.

It is important to note that this pilot study was conducted on tissues sampled during autopsy. This represents the strength of this study. Thanks to the post mortem findings, all groups were composed of subjects who had died due to an exact cause of death. Particularly, referring to the AAS and CKD groups: on the one hand, in the AAS group, we enrolled subjects that certainly had used only these substances, with negative toxicological tests for other illicit drugs; on the other hand, in the CKD group, subjects who had suffered for a long period of time (from 2 to 5 years) from Chronic kidney disease were selected. Moreover, we compared the expression values of the selected miRNAs with the data obtained from the control group that was composed of healthy subjects who had died from traumas after a car accident.

The main limitation of this study is related to the fact that the AASs use was ascertained through a toxicological examination, even if data about the exact duration of use is unknown. Another significant limitation is related to the small number of subjects enrolled in the AAS group. Concerning this last consideration, it is important to highlight that the number of subjects who die with a positive toxicological test for anabolic-androgenic steroids is low in Italy: for example, in our Institute, we found only 7 cases analyzing 1,700 autopsy records from the last 15 years. Finally, it was unclear if the abnormalities, particularly for AAS users, were premorbid (chicken-and-egg problem).

## Conclusions

In the last few years, miRNAs have been considered as very promising bio-signatures both to identify human pathologies earlier and to improve the outcome of patients with specific drug therapies. Nowadays, thanks to modern technologies, this kind of approach has become very promising in all fields of medicine (Bader and Lammers, 2012; Neudecker et al., 2016; Kreth et al., 2018; Bertozzi et al., 2019a).

Another important problem for developed countries is the increasing number of elderly people. Moreover, an aged-population has different health problems that can cause degenerative kidney diseases, increasing hospitalization, and consequently, generating a high cost for public health management. In this scenario, this experimental study aimed to analyze the role of miRNAs in kidney injury after AAS use, to better understand the underlying regulating mechanisms. The results highlighted that the investigated miRNAs presented different expression patterns in kidney disease, suggesting a pivotal role. As described above, miR-21 and miR-205 were expressed significantly higher both in the AAS and the CDK groups compared to the Control group, suggesting a cumulative role in adverse kidney injury effects, promoting fibrosis. For these reasons, they could be evaluated as molecular biomarkers of renal damage. On the other hand, the analyses performed showed a marked increase in the expression of miR-21 in AAS users compared to miR-205. It is difficult to say, based on the data currently available, if an under-expression in some cells involved in a tumor transformation contribute to the overall tissue value of miR-205. Therefore, if the promoter pathway of fibrosis can be considered, any conclusion on the possibility of tumor development induced by AAS consumption should be considered inconclusive. Moreover, this study represents a pilot study to define if these expression patterns could be studied in other biological samples (plasma, urine) in patients with different kidney injury linked to chronic kidney diseases and AAS use, to identify reliable biomarkers that could be applied in clinical and forensic diagnostics, as well as a target for toxicological investigations or therapeutic treatments. In the near future, confirming the plasma expression alteration, these miRNAs could represent reliable biomarkers to quickly diagnose similar damage, or alternatively, they could represent the target of new drugs based on the inactivation of the activities of these miRNAs. In light of these findings, further studies with larger samples are needed to confirm these interesting findings.

## Data Availability Statement

All datasets presented in this study are included in the article/**Supplementary Material**.

## Ethics Statement

The studies involving human participants were reviewed and approved by Scientific Committee of the University of Foggia. Informed consent was obtained from the relatives.

## Author Contributions

FS, LC, GB, and CP conceived and planned the experiments. FS, GM, ET, and MS carried out the experiments. FS, LC, MS, MA, ET, GB, and CP contributed to sample preparation. FS, MS, GB, GM, and CP contributed to the interpretation of the results. FS and GB performed the statistical analysis. FS and CP. wrote the manuscript in consultation with GB and MS. All authors contributed to the article and approved the submitted version.

## Conflict of Interest

The authors declare that the research was conducted in the absence of any commercial or financial relationships that could be construed as a potential conflict of interest.

All authors are affiliated to public University with the exception of LP that is affiliated with a private society (Brain srl, Services and consultancy in health, 27100 Pavia). This society does not operate in the same field of the publication: for this reason, it did not influence the study design, execution, analysis or decision to publish.
