# Supplementary material for: miRNAs as Novel Biomarkers of Chronic Kidney Injury in Anabolic-Androgenic Steroid Users: An Experimental Study
Source: Front Pharmacol. 2020 Sep 16;11:563756. doi: 10.3389/fphar.2020.563756 (PMC7525215; doi:10.3389/fphar.2020.563756)
Supplement: Supplementary file 1 [file Table_1.docx]

**TABLE**

|  | | **Expression levels**  **(endogenous control: miR-186)** | | **Expression levels**  **(endogenous control: miR-361)** | |
| --- | --- | --- | --- | --- | --- |
|  |  | **miR-21-5p** | **miR-205-5p** | **miR-21-5p** | **miR-205-5p** |
| **AAS**  **Group** | **S1** | 2.77± 0.02 | 0.98± 0.015 | 2.67± 0.015 | 0.91± 0.015 |
|  | **S2** | 1.32± 0.01 | 0.39± 0.011 | 1.41± 0.011 | 0.51± 0.02 |
|  | **S3** | 0.96± 0.05 | 0.15± 0.05 | 1.19± 0.053 | 0.21± 0.015 |
|  | **S4** | 2.36± 0.06 | 1.51± 0.07 | 2.21± 0.011 | 1.65± 0.014 |
|  | **S5** | 0.73± 0.05 | 1.33± 0.01 | 1.29± 0.05 | 0.91± 0.015 |
|  | **S6** | 1.5± 0.04 | 0.59± 0.06 | 1.69± 0.02 | 0.52± 0.017 |
|  | **S7** | 1.95± 0.05 | 0.44± 0.05 | 2.15± 0.06 | 0.63± 0.05 |
| **CKD**  **Group** | **S1** | 2.33± 0.02 | 0.9± 0.01 | 2.6± 0.05 | 1.11± 0.015 |
|  | **S2** | 3.77± 0.06 | 1.7± 0.02 | 3.43± 0.01 | 1.92± 0.017 |
|  | **S3** | 3.23± 0.02 | 2.8± 0.04 | 3.32± 0.06 | 3.02± 0.01 |
|  | **S4** | 3.45± 0.05 | 1.2± 0.023 | 3.64± 0.01 | 1.3± 0.05 |
|  | **S5** | 2.82± 0.05 | 0.79± 0.025 | 2.61± 0.015 | 0.98± 0.06 |
|  | **S6** | 1.89± 0.06 | 1.5± 0.015 | 2.03± 0.011 | 1.41± 0.015 |
|  | **S7** | 2.3± 0.02 | 2.59± 0.041 | 2.3± 0.01 | 2.31± 0.02 |

**Table S1**. Expression levels of miRNAs analyzed in each sample.
